# Impact of participation in continuing medical education small group learning (CME-SGL) on the stress, morale, and professional isolation of rurally-based GPs: a qualitative study in Ireland

**DOI:** 10.3399/bjgpopen19X101673

**Published:** 2019-10-30

**Authors:** Stephanie Dowling, Jason Last, Henry Finnegan, Pat Daly, John Bourke, Conor Hanrahan, Pat Harrold, Geoff McCombe, Walter Cullen

**Affiliations:** 1 ICGP Assistant National Academic Director of CME, Irish College of General Practitioners, Dublin, Ireland; 2 Research Student, Health Sciences Centre, University College Dublin School of Medicine, Dublin, Ireland; 3 Associate Dean, Director of Education Development and Academic Affairs, University College Dublin School of Medicine, Health Sciences Centre, University College Dublin, Dublin, Ireland; 4 Former National Director of ICGP CME (retired), Irish College of General Practitioners, Dublin, Ireland; 5 ICGP CME Tutor, Irish College of General Practitioners, Dublin, Ireland; 6 ICGP CME Tutor, Irish College of General Practitioners, Dublin, Ireland; 7 ICGP CME Tutor, Irish College of General Practitioners, Dublin, Ireland; 8 ICGP CME Tutor, Irish College of General Practitioners, Dublin, Ireland; 9 Post-doctoral Research Fellow, University College Dublin School of Medicine, Health Sciences Centre, University College Dublin, Dublin, Ireland; 10 Professor of Urban General Practice and Head of Subject, General Practice, University College Dublin School of Medicine, Health Sciences Centre, University College Dublin, Dublin, Ireland

**Keywords:** continuing professional development, education, medical, continuing, postgraduate education, rural medicine, general practice, primary health care, family practice, general practitioners, burnout, professional

## Abstract

**Background:**

The pressures of general practice contribute to high levels of stress, low morale, and burnout in some GPs. In addition, rurally-based doctors may experience significant professional isolation. Participation in continuing medical education (CME) appears to reduce stress, and may improve the retention of rural GPs.

**Aim:**

As part of a larger study devised to examine the effectiveness of regular participation in CME small group learning (SGL) on rurally-based Irish GPs, this study explored whether CME-SGL had any impact on GP stress, morale, and professional isolation.

**Design & setting:**

This was a qualitative study involving four CME-SGL groups based in rural Ireland.

**Method:**

Semi-structured focus group interviews were conducted in established CME-SGL groups in four different rural geographical locations. Interviews were audiorecorded, transcribed verbatim, and analysed thematically.

**Results:**

All members of these CME-SGL groups (*n* = 43) consented to interview. These GPs reported that regular meetings with an established group of trusted colleagues who are ‘in the same boat’ provided a ‘safe space’ for discussion of, and reflection on, both clinical concerns and personal worries. This interaction in a supportive, non-threatening atmosphere helped to relieve stress, lift morale, and boost self-confidence. The social aspect of CME-SGL sustained these rural GPs, and served to alleviate their sense of professional isolation.

**Conclusion:**

Delivery of CME through locally-based SGL provides as an important means of supporting GPs working in rural areas. The non-educational benefits of CME-SGL, as described by these Irish GPs, are of relevance for rural doctors in other countries.

## How this fits in

It is known that participation in CME supports doctors and helps to reduce stress. Access to CME may also reduce professional isolation and improve the retention of rural GPs. This study is the first to examine the effects of participation in locally-delivered CME through SGL on the stress levels, morale, and professional isolation of Irish GPs working in rural locations. The finding that there are a number of non-educational benefits of CME-SGL which support and sustain participants is of relevance for rural doctors elsewhere, and therefore CME-SGL may be a support for rural single-handed GPs who are more professionally isolated.

## Introduction

The pressures facing GPs are higher than ever and continue to increase, driven by escalating bureaucracy, increased patient demands, workforce shortages, and a reduction in resources.^1–6^ It is argued that these pressures have contributed to low job satisfaction and low morale among staff, as well as stress and burnout.^7^ The emotional component of work in general practice is also known to impact on the personal wellbeing of GPs.^8^ Workload and stress influence job satisfaction, which, in turn, is the main predictor of retention of GPs.^9^ Access to CME has been identified as being of key importance as a support for doctors in their work, and may help to reduce the stress levels of GPs.^10–13^

Research suggests that professional isolation (from colleagues, consultants, and locums) and access to CME are key factors affecting recruitment and retention for rural GPs^13^. The World Health Organization, as part of a policy to improve retention of rural healthcare workers, recommended that governments design CME programmes that meet their needs.^14^ A number of studies report that, compared to urban GPs, rural physicians perceive that their opportunities for participation in traditional CME activity are inadequate.^12,15–17^

In the Republic of Ireland, CME for GPs is delivered by a network of 37 Irish College of General Practice (ICGP) tutors, each of whom coordinates SGL educational sessions for approximately three to six groups of doctors (usually 8–12 members). Meetings are held in the evenings and last approximately 2 hours; each group typically has up to eight meetings per year. The tutor network covers all of the Irish Republic, with SGL occurring in both urban and rural locations, so that all GPs are able to access CME-SGL locally. Group leaders (who are themselves group members) facilitate teaching under the direction of the tutors; participants discuss cases, reflect on evidence presented in the meeting, and consider what changes they will make to their own practice. A recent study found that the educational needs of Irish GPs are met by ICGP CME-SGL.^18^

Structured small group work such as Irish CME-SGL, Balint groups, or similar continue to be employed in general practice and are valued by GPs.^19^ SGL is highly valued as an effective method of CME in comparison to traditional methods such as lectures.^20^ What constitutes a ‘small group’ depends on cultural context, and the terms ‘peer review group’, ‘quality circle’, ‘CME group’, ‘practice-based small group work’, and ‘small group work’ are used interchangeably in different European countries.^21^

Riley and colleagues^6^ reported that GPs who had greater collegial supports feel less isolated, more resilient, and better able to cope with the emotional and clinical demands of their work. A recent Irish study found that GPs who attend CME-SGL may have higher morale and lower stress levels than GPs nationally.^18^ The study reported here was devised to explore the impact of attendance at CME-SGL on the stress levels, morale, and professional isolation of Irish GPs who practice in rural locations.

## Method

### Design

The authors conducted semi-structured GP focus group interviews within established rurally-located Irish CME-SGL groups with the intention of exploring the impact of CME-SGL on doctors and their patients. The topic guide used for the interviews was informed by themes emerging from a previous Irish study;^18^ however, discussions were allowed to occur naturally within each group.

### Participants and recruitment

GPs were recruited from four CME-SGL groups run by different tutors based in different rural geographical locations in the Republic of Ireland in order to serve as a representative sample of rural Irish GPs. Any GP attending the CME-SGL group could participate; there was no financial reimbursement. Consent was obtained for participation and for the researcher to sit in on the groups during the semi-structured interviews.

### Data collection

The four 30-minute focus group interviews — each facilitated by the primary researcher, who is also a GP — took place in October and November 2018. Topics covered (Box 1) were how CME in this format (that is, CME-SGL) impacted on clinical practice and patient outcomes, and whether it had any impact on stress, morale, or professional isolation. A semi-structured approach to data collection was adopted so that topics or issues that arose in the focus groups could be explored. Debate and drawing out of issues was encouraged. Interviews were recorded and transcribed. The two lead researchers independently reviewed all four interviews and, following debate, established the point of data saturation (when no new themes of interest were emerging).^22^

Box 1 **Interview topic guide**

- Why do GPs use CME-SGL compared to other methods of learning?
- How practical is it for you to use this form of learning?
- How does CME impact on healthcare outcomes for patients?
- Does CME-SGL deal with GP stress?
- Does CME-SGL affect GP morale?
- How does CME impact on the healthcare system?
- Does it help with professional isolation?
- Is it likely to affect recruitment and retention?

CME = continuing medical education. SGL = small group learning.

### Analysis

Coding was data driven according to the grounded theory approach described by Charmaz,^23^ and was agreed by the research team. The first stage involved open coding of all transcripts into main themes, while the second stage involved categorisation of the themes into sub-themes. All four transcripts were coded and completed by two authors. Once data was collected and coded, another member of the team independently coded two transcripts to ensure coding was accurate. Field notes, memos, coding, and theoretical development were discussed at regular team meetings. NVivo (version 12) was used for data management.^24^

## Results

All GPs (*n* = 43) in four CME-SGL groups based in rural areas in Ireland consented to be involved in the study (Table 1). Just under two-thirds of these GPs self-identified as being in rural practice (*n* = 27); another 12 worked in mixed practices (that is, had responsibility for two centres of practice, one urban and one rural). Thus, overall, 39 GPs (90.7%) worked exclusively or partly in rural practice. Only four GPs worked in exclusively urban practices, but participated in rurally-based CME-SGL. Of the 27 GPs working solely in rural practice, 17 (63.0%) were single-handed. Rurally-based GPs were more likely to be male (56.1%), attending CME-SGL for longer (13 years), and in practice for more years (19 years).

**Table 1.** GP experience (in years) of four CME-SGL groups

	**Group 1**	**Group 2**	**Group 3**	**Group 4**
GPs in the group, *n*	10	11	11	11
Practice type of GPs in the group	Rural 6/Mixed 2/Urban 2	Rural 10/Mixed 1	Rural 8/Mixed 3	Rural 3/Mixed 6/Urban 2
Male GPs, *n* (%)	4 (40.0%)	6 (54.5%)	7 (63.6%)	6 (54.5%)
Group total number of years attending CME (average years per GP)	114 (11)	113 (11)	169 (15)	130 (12)
Group total number of years working as a GP (average years per GP)	168 (17)	157 (14)	232 (21)	272 (25)

CME = continuing medical education. SGL = small group learning.

Discussions in focus groups developed easily and, once the facilitator raised a topic from the interview guide (Box 1), minimal additional facilitation was required. The broad themes of GP stress, morale, and professional isolation were explored, with particular focus on whether CME-SGL is helpful, and if so, how.

### Stress

GPs reported that they felt their colleagues in the CME-SGL groups were able to support them as they all did the same job, and so could understand their concerns and daily challenges:

*‘* *I think also there’s a natural therapy being surrounded by people in the same situation as you. They really understand what you do every day …* *’ (* *GP4)*

*‘I* *think as well as maybe discussing issues that might stress us, I think it’s useful to know that we all have the same problems … more or less the same problem patients … the same management problems … you think … the whole world is against me … you come in and meet the others and … well, it’s not just me.* *’ (GP3)*

Most of these GPs had been members of their respective CME-SGL groups for several years. They reported that the trust that had been established in the group was an important factor in their ability to discuss difficulties they were experiencing. They felt safe bringing up confidential topics for discussion in the group:

*‘* *Well, most of us are single-handed, you know, and who do you turn to? You can’t bring it home … I definitely find it very supportive here … the longer we go on, the more you build up the trust in the group … and it’s extremely helpful. It’s good to know that you’re not on your own in difficult situations when you might be a bit vulnerable yourself.* *’ (GP2)*

*‘* *Well, there are certain things you could bring up in the forum of the meeting because it is confidential … we all know each other for a long time now because it’s the same group.* *’ (GP1)*

These GPs felt able to discuss mistakes or worries they were experiencing within a supportive group. The ability to obtain advice from experienced colleagues was viewed as important, particularly for GPs starting out in their careers in general practice:

*‘Where* *we did make mistakes, just the whole experience of it and the stress of it and the difficulty … bringing it up at CME is a worthwhile experience. And also … experienced GP* *s do have a lot to offer … I certainly feel I’m a lot better now than when I was 28 …* *’ (* *GP3)*

*‘* *We have a young GP who qualified last year … she really loves the CME group and she finds them so supportive and helpful … it’s more than just the education I think for a young practitioner starting out. What’s important for that age group might be a little bit different from those of us established in practice.* *’* *(GP3)*

The social side of CME-SGL was highlighted as an important part of being able to discuss their concerns. GPs knew they could relax in the group, and felt that it was a safe place to chat about their work:

*‘* *There’s a social element to it as well … there are colleagues you wouldn’t see from one end of the year to another if you weren’t coming to CME … from a stress and a mental health point of view that’s great because you know you can thrash out things … I think that’s a very important side of it as well. You have a cup of tea afterwards and chat.* *’* *(GP1)*

*‘* *A lot of the actual thing is the value of a group of GPs getting together.* *’* *(GP2)*

### Morale

The view was expressed that the discussions that occurred within the CME-SGL groups among colleagues who are doing the same job helped to reinforce good clinical practice. GPs reported that they found this supportive, and that it boosted their confidence in their work:

*‘* *I guess we all have the same sort of practices … you learn from your colleagues … you feel that you’re maybe not doing so badly if everybody else is, you know, the same. So it helps your confidence in some ways.* *’* *(GP2)*

*‘* *It makes you more confident in doing what you do because you know you’re not the only one doing such and such, that everyone seems to be on the same level and they’re doing whatever you happen to be doing as well … it reinforces good practice.* *’* *(GP4)*

The non-threatening atmosphere at CME-SGL was felt to be very important. GPs felt able to be completely honest about how they actually manage patients, and felt that they could discuss their concerns and worries openly in a supportive place. The atmosphere was supportive; GPs felt able to bring up certain areas for discussion with their colleagues which might otherwise be difficult to address, in particular when they were going through tough times, both professionally and personally:

*‘* *I think it’s a very accessible sort of forum and I find that the exchange between each of the individuals at all the meetings is very worthwhile. There are no axes being ground here, and … the atmosphere is very pleasant. It mightn’t be the topic for discussion that’s the learning point. You might just discuss a case or a difficulty you’re having … you know if you learn one thing at a meeting it’s probably a good day’s work.* *’* *(GP2)*

*‘* *I like the comradery, and the atmosphere in the group is excellent … I always learn something. I know we’ve gone through a few bad years … with the financial cuts and rural depopulation … that was all aired in CME at certain stages, and the frustrations with the job … then there were difficulties with suicides in the area … all these kind of issues are brought up.* *’* *(GP2)*

### Professional isolation

Professional isolation was a common theme discussed across all of these CME-SGL groups, and occurred both in single-handed and group practices. These GPs reported that they considered CME-SGL to be an important outlet which helps to alleviate such isolation:

*‘* *Sometimes you feel like you are very isolated in general practice even when you’re in a group practice …* *’* *(GP4)*

*‘* *It’s certainly a very positive and worthwhile ingredient of the whole medical, social, collegiate endeavour. You don’t have to feel that you’re practicing in isolation because you meet 10 or 15 people here who’ve all got individual little dilemmas and we get an opportunity to air them and somebody else will throw in a helpful comment and so forth. You mightn’t be able to back it up with evidence-based findings, but it can be very helpful.* *’* *(GP2)*

GPs felt that attendance at CME-SGL allowed them to compare their practice to colleagues in a similar position to see how they are doing. CME-SGL forced them to reflect on their own approach, and the active learning which took place at meetings served to reinforce best practice:

*‘* *I think it can be very reassuring … when you come to the group and you hear what everybody’s doing if* *… you’re doing similar things … you’re very isolated sometimes, doing your own thing especially as a single-handed GP, or if you’re job sharing you might be on your own opposite the other person* *.* *’* *(GP1)*

*‘* *You’re ploughing your own furrow every day but every month or so you’re forced to reflect on what you do, what you accept as standard practice. You’re forced to think about the simple things.* *’* *(GP4)*

The professional isolation of single-handed GPs limits their opportunities to discuss stressful experiences or events. These GPs reported that CME-SGL plays an important role in this respect by providing a forum for such discussions:

*‘* *I’m in single-handed practice so I don’t have the experience … of having doctor colleagues on a day-to-day basis. There is no other forum for discussing mistakes or stressful events or sudden deaths … or situations where you feel culpable and you may not have been culpable. But you can’t unwind those thoughts … I certainly think that CME has a big role to play.* *’* *(GP4)*

*‘* *You know, it would definitely be something that would help sustain me in my practice.* *’* *(GP2)*

In summary, the GPs who participated in these focus groups, most of whom work in rural locations, reported that CME-SGL helped to sustain them in their jobs, and to cope with difficulties they experienced in their day-to-day work.

## Discussion

### Summary

The GPs who participated in this study, almost two-thirds of whom practiced in rural locations, reported that CME-SGL supported them in their work. The possibility to meet locally each month with colleagues who faced the same work, concerns, and challenges, along with the mutual trust established over a number of years, afforded a safe space for GPs to bring up confidential issues, including mistakes made in practice, along with more personal worries. This, in turn, was important for the relief of stress and lifting of morale. The supportive, non-threatening, reflective atmosphere, along with the experience of colleagues — some of whom have been in practice for decades — was considered important for reinforcement of good practice, particularly for younger, less experienced GPs, and for boosting of self-confidence for established GPs. The social aspect of CME-SGL was especially valued; rural GPs felt sustained by the monthly meetings, which served to alleviate their sense of professional isolation. These non-educational benefits of CME-SGL are of relevance for rural doctors in other countries, and for all health services struggling with recruitment and retention of rural GPs.^25,26^

### Strengths and limitations

In this study, 27 of the 43 GPs surveyed self-identified as being rural practitioners, while a further 12 had responsibility for both rural and urban practices. Of the rural practitioners, 17 were working alone in single-handed practice. In Ireland, the Health Service Executive designates certain GP practice areas as rural for the purposes of a dedicated payment (rural practice allowance);^27^ accordingly, it is considered that these GPs were able to accurately identify that they worked in a rural practice. These doctors were more likely to be male, and in practice for longer, findings which are consistent with other major Irish surveys of rural doctors,^2,25^ and which suggest that the sample selected for this study is representative of rural GPs. In Ireland, rural practitioners have a scattered patient population, have few colleagues located nearby, and are situated at a distance from most referral facilities.^25^ It is acknowledged that not all study subjects worked exclusively in rural locations; moreover, because of the size of Ireland, relatively few GPs in rural practice are extremely isolated. Nevertheless, 90.7% of participants worked wholly or partly in rural practice, suggesting that the study conclusions can reasonably be applied to other rural GPs. That said, the extent of diversity between international primary care structures may limit the generalisability of the current findings to other cultural contexts.

The challenge of controlling for bias in this qualitative study with a relatively small sample was met in three ways. Firstly, all GPs in the naturally occurring CME-SGL groups were invited to participate, and all accepted. The 100% response rate ensured that each focus group was likely to be representative. Secondly, four different focus groups with different tutors and from different (rural) geographical locations were selected, minimising the chance that local issues might affect the results, while increasing their diversity and strengthening their representativeness. Thirdly, the focus groups were conducted at the usual CME-SGL meeting locations, with the primary researcher, who is also a GP, travelling to sit in on the discussions.

It is conceded that qualitative methodology used in this study is exploratory in nature, and so better suited to provide insight into how and why CME-SGL may impact on stress, morale, and professional isolation, rather than to confirm whether it has an impact or not. The use of long-existing CME-SGL groups facilitated robust interaction during these discussions; however, it is acknowledged that established group dynamics could have influenced the input of some participants. A disadvantage of group interviewing is that there may be individuals who do not feel able to speak up in this environment; for example, some participants may be unwilling to report negative aspects, and younger doctors may feel that they can’t contribute as much as their more experienced colleagues on a particular topic raised. In this case, the primary researcher was present at all focus groups; recorded field notes confirm that all GPs participated in the interviews, with younger GPs contributing just as much as older GPs to the discussions.

Although clinician researchers have been shown to get richer data from GP participants than non-clinical researchers, this can introduce clinical bias into the data collect and interpretation.^28^ In this study, the risk of professional bias was addressed by including researchers with diverse professional backgrounds on the research team.^29^ The sample size was likely sufficient given that data saturation, as established by the lead researchers, was achieved.^30^

### Comparison with existing literature

SGL provides opportunities for rural physicians to meet with colleagues and have time away from practice obligations, incorporating personal, social, and professional experiences into the learning process.^31^ Such groups provide a safe and supportive space where doctors can openly discuss the pressures and emotional challenges of work; this, in turn, provides GPs with the support they need while offering protection against compassion fatigue and burnout.^32^ Collegial support is a protective factor for good mental health, and is associated with resilience and reduced sickness.^33^ A Danish study found that GPs who were not active members of a CME group had a higher likelihood of suffering from burnout that those who participated in such a group.^34^ A systematic review on interventions to reduce burnout among doctors found that improving communication and giving participating doctors permission to acknowledge and manage stress have proven effective.^35^ The finding in the current study that CME-SGL reduces the stress levels of rurally-based Irish GPs is directly in line with the above.

A study conducted in the Northern Ireland and the Republic of Ireland found that older GPs who were in practice for >20 years have better morale and lower stress than younger colleagues.^36^ The authors suggested that the experience of older GPs was an untapped resource that could find useful expression in mentorship of their younger colleagues. Gardiner *et al* ^37^ aimed to improve psychological wellbeing of rural GPs through a programme that included formation of a peer support network of experienced colleagues whom these GPs could access. Following introduction of this network, GPs reported increased levels of social support and lower levels of distress compared to baseline. The ICGP CME-SGL structure may act as peer support network; older GPs who participated in this study specifically identified CME-SGL as a source of support for each other and, in particular, for their younger colleagues.

Huby *et al* ^38^ suggested that any measure to improve morale in general practice will have to enable GPs to express and deal with the complexity of primary care, including concerns about workload, practice issues, and personal issues. When rural GPs were asked which skills they would most like to acquire to assist them in rural practice, nearly 40% of the responses related to the need for personal coping skills rather than clinical skills.^39^ Gardiner and colleagues reported that the more contact GPs have with each other (that is, the more support they receive), the better their work-related morale and quality of life, and the lower the level of their distress. Moreover, interaction with colleagues may be of benefit to GPs who are considering leaving rural practice and may increase the number who ultimately stay in rural practice.^39^ The current study found that regular interaction with colleagues whom they have known for a long period of time in a supportive, non-threatening atmosphere at CME-SGL helps to sustain rural GPs in their work. Morale is boosted through reinforcement of good clinical practice, improved confidence, and by providing a forum in which GPs can discuss their concerns and worries openly.

White *et al* ^12^ studied CME workshop attendees by questionnaire over a 3-year period (429 responders) and found that 94% agreed or strongly agreed that access to CME contributed to confidence in practicing in rural and/or remote locations. Of these GPs, 93% agreed or strongly agreed that access to CME alleviated professional isolation, and 80% agreed or strongly agreed that they were less likely to remain in rural practice without such access.^12^ Although large, this quantitative study was not able to explore these findings in more detail. In contrast, by using qualitative methods, the current study was able to explore the impact of CME-SGL on stress, morale, and professional isolation of rurally-based GPs in more depth and with fewer responders. In another qualitative study of similar size (47 participants), the authors found that GPs, particularly those working alone in single-handed practice, feel less isolated where CME offers the opportunity to meet with colleagues.^6^ Riley concluded that providing a safe space for GPs to process the emotional and clinical content of their work is imperative. This is consistent with the current study which found that participation in CME-SGL is an important support for GPs in preventing professional isolation.

The use of e-learning is considered to represent a promising method to support the CME needs of rural GPs, and a number of distance learning programs have indicated satisfaction with such initiatives among participants.^40^ Despite this, a recent review focused on CME for rural GPs concluded that distance learning initiatives did not impact on doctor performance or patient outcomes.^21^ This may be due to poor uptake of internet-delivered CME. In a study of an online diabetes educational module, Paul *et al* found that of 34 (23.3%) GPs who enrolled on the programme, only 8 (5.5 %) completed it.^41^ In contrast to e-learning, SGL provides opportunities for rural physicians to meet with colleagues and have time away from practice obligations, incorporating personal, social, and professional experiences into the learning process.^31^ This, along with the positive findings seen in this study for stress, morale, and professional isolation, may be why the majority of GPs in Ireland use CME-SGL in preference to other methods available.^42^

### Implications for research and practice

One of the negative factors that makes rural primary care a less attractive proposition for GPs is the prospect of working alone without any backup from colleagues.^43^ In Ireland, the number of single-handed practices, which are much more commonly located in rural areas, is in continual decline, having reduced from 62% in 1982 to 18% in 2015.^2^ The same trend has been seen in the UK, where <10% of GPs work in rural practice.^44^ The situation elsewhere in Europe is similar.^45^ Rurally-based doctors suffer from loneliness and personal isolation; in addition, both rural GPs and single-handed GPs frequently feel professionally isolated.^26,44^ GPs working in single-handed practices tend to suffer from higher stress levels than those who don’t.^46^ The results of this study suggest that locally based CME-SGL is a possible part of the solution to supporting professionally isolated GPs, and may have applicability to rural doctors in other parts of the world.

While the recruitment and retention of rural physicians is influenced by a variety of factors, including personal history (such as rural background), family issues (spouse and children), educational factors (for example, rural exposure during medical training), community lifestyle and integration, and professional, economic, and organisational factors, it is recognised that access to CME is of key importance.^13,47,48^ This study reports some of the reasons why locally-delivered CME-SGL is highly valued by GPs engaged in predominantly rural practice. The benefits of regular contact with a small group of colleagues through attendance at locally-based CME meetings go well beyond the education received. There are clear gains in wellbeing, including relief of stress, boosting of morale, and alleviation of professional isolation. This is of relevance because, aside from benefits for recruitment and retention of rural GPs, there is evidence of a clear link between the wellbeing of doctors and the experiences of their patients.^49^ It is proposed that this method of educational delivery should be seen as an important means of supporting GPs in rural areas, and may help to retain GPs in otherwise isolated practices. In the words of one focus group participant:

*‘* *I think that the government needs to … see this as a good support for doctors who really have very few other supports in what is an extremely difficult job.* *’ (GP4)*
